# Long-Term Effectiveness of Antiretroviral Therapy in China: An Observational Cohort Study from 2003–2014

**DOI:** 10.3390/ijerph120808762

**Published:** 2015-07-24

**Authors:** Peng Huang, Jingguang Tan, Wenzhe Ma, Hui Zheng, Yan Lu, Ning Wang, Zhihang Peng, Rongbin Yu

**Affiliations:** 1 Department of Epidemiology and Biostatistics, School of Public Health, Nanjing Medical University, Nanjing 211166, China; E-Mails: hp19880310@163.com (P.H.); mawenzhe7@njmu.edu.cn (W.M.); zhenghui8946@hotmail.com (H.Z.); 2 Shenzhen Center for Disease Control and Prevention, Shenzhen 518055, China; E-Mails: tangjingguang2014@126.com (J.T.); luyan_sz@126.com (Y.L.); 3 National Center for AIDS/STD Control and Prevention, Chinese Center for Disease Control and Prevention, Beijing 102206, China; E-Mail: wangning_12@126.com

**Keywords:** HIV, ART, mortality, observational cohort study, China

## Abstract

In order to assess the effectiveness of the Chinese government’s expanded access program, a cohort study on all adult HIV patients in Shenzhen was conducted from December 2003 to February 2014 to estimate the effects of antiretroviral therapy (ART) on mortality, tuberculosis and CD4 cell counts. Marginal structural regression models adjusted for baseline and time-varying covariates. Of the 6897 patients enrolled and followed up for a maximum of 178 months, 44.92% received ART. Among patients who commenced receiving ART during the study, there were 98 deaths and 59 new tuberculosis diagnoses, while there were 410 deaths and 201 new tuberculosis diagnoses among those without ART. ART was associated with both lower mortality (hazard ratio [HR] = 0.18; 95% confidence interval [CI] = 0.11–0.27) and the presence of tuberculosis (HR = 0.27; 95% CI = 0.19–0.37). Each month of ART was associated with an average increase in CD4 cell count of 6.52 cells/µL (95% CI = 6.08–7.12 cells/µL). In conclusions, the effectiveness of ART provided by China government health services is the same as that in higher-income countries. Accounting to higher mortality rates from the delay of starting ART, faster expansion and timely imitation of ART are urgent.

## 1. Introduction

In China, a cumulative 436,817 people living with HIV/Acquired Immune Deficiency Syndrome (AIDS) had been identified as of December 2013, including 173,825 people with AIDS [1,2]. A remarkable acceleration in treatment has occurred since the China National Free Antiretroviral Treatment Program (NFATP) was established in 2003 [3,4]. By December 2013, antiretroviral therapy (ART) had been given to more than 278,000 people. Along with the growing treatment coverage, overall mortality rates have fallen from 39.3 deaths per 100 person-years in 2000 to 14.2 deaths per 100 person-years in 2009 [5,6]. Many previous studies in China describe improved outcomes among patient cohorts receiving ART, but fewer studies have compared results between patients with ART and similar patients without it [7,8]. Without treatment, health outcomes tend to deteriorate, and a complete picture of ART’s effectiveness may not be available by describing changes in outcomes only among patients with ART. Moreover, since treatments are largely selected according to patients’ health status, comparisons of treated and untreated patients are likely to be biased [5,9]. Randomized placebo-controlled trials can provide valid estimates, but in ART studies they are unethical [10]. However, successive observations of changes in individuals have recently been performed in cohort studies from Europe and South Africa, and it is possible to adjust for these selection biases [11,12].

Since time-dependent confounders are affected by treatment, marginal structural models adjusted for time-varying CD4 cell counts can avoid selection bias [11]. This study followed up all people at least 18 years of age with an HIV infection in Shenzhen from December 2003 to February 2014 and estimated the effects of ART on mortality, tuberculosis and CD4 cell counts by unweighted and weighted models.

## 2. Methods

### 2.1. Patients and Treatment Regimens

We conducted a cohort study with repeated measures. The study population comprised all people at least 18 years of age with an HIV infection in Shenzhen from December 2003 to February 2014. Patients were mainly diagnosed and then included into this study from three ways. Firstly, patients were self-present when they had high risk behaviors. In addition, they were tested when presenting for other illnesses in hospital. Last, a part of patients came from HIV sentinel surveillance, in which high risk populations such as drug users and men having sex with men (MSM) were tested every year. Patients with fewer than two recorded program contacts were excluded from the study. In accordance with Chinese policy, ART was given to all HIV-positive patients who met the national treatment guidelines of a CD4 cell count less than 350 cells/µL (<200 cells/µL before 2008) or World Health Organization (WHO) stage 3 or 4 disease [13]. Patients with active tuberculosis or other serious opportunistic infections could not receive ART until they were clinically stable or until the intensive phase of tuberculosis treatment was completed. This study was approved by the Institutional Review Board of Nanjing Medical University. Written informed consent was obtained from each participant after a full explanation of the study.

Treatment procedures were conducted in accordance with the Chinese National Free HIV Antiretroviral Treatment Guidelines [13]. The first-line regimen was a combination therapy of two nucleoside reverse transcriptase inhibitors (NRTIs) and one non-nucleoside reverse transcriptase inhibitors (NNRTIs), which consisted of lamivudine (3TC), zidovudine (AZT), stavudine (d4T), didanosine (ddI), tenofovir (TDF), nevirapine (NVP), and efavirenz (EFV). After the national antiretroviral treatment guidelines were updated in 2008, ART failure participants were generally switched to a second-line regimen, which included TDF and lopinavir/ritonavir (LPV/r).

### 2.2. Data Collection

Patients receiving ART were monitored at two weeks; one, two and three months; and once every three months thereafter. CD4 cell counts were measured at enrollment and every three months thereafter for those with receiving ART or every six months for those not receiving ART. Viral loads and WHO stages were recorded for patients receiving ART [13]. 

Patient information consisted of demographic characteristics, laboratory results, and current symptoms. Survival time was defined as extending from the date of enrollment or ART initiation until death or the date of the last follow-up. For mortality cases, detailed information was tracked from the *China Disease Surveillance Information Reporting System*. The database contains information of patients on demographic characteristics, survival time (from diagnosis to death), cause of death, and so on. Moreover, any patient missing four consecutive follow-up appointments was identified as lost to follow-up, and the date of the last follow-up was regarded as the termination date.

Pulmonary tuberculosis was primarily diagnosed by examining sputum smears, in accordance with national guidelines [3]. Physicians diagnosed extra-pulmonary or smear-negative pulmonary tuberculosis, taking into account other clinical or radiologic information [13].

### 2.3. Statistical Analysis

A two-phase analysis was performed: A pooled logistic regression and a marginal structural mode. We collected and summarized the baseline information, including age, sex, location, baseline CD4 cell count, tuberculosis status, AIDS stage, and time-varying covariates at latest CD4 cell count. A marginal structural model was applied to accurately demonstrate the relationship between explanatory and outcome variables. Explanatory variables were ART status and per additional month of ART; outcomes identified in our study included death, CD4 cell count and newly diagnosed tuberculosis. Once a patient started ART, the binary variable of ART status was assumed to be 1. 

The inverse probability of the explanatory variables, the key point of the marginal structural mode, was obtained by two different logistic regression models. The first one involved only the baseline variables, and the second one included these with the time-varying variables. The probability weights and the proxy of the influence of covariates were fitted in a logistic regression with death and tuberculosis, and in a linear regression with CD4 cell count. In our analysis, mortality was censored at death or at last follow-up, and tuberculosis status was censored at the time of diagnosis or at the last recorded time. 

Because a logistic regression requires that the subjects be removed from the population at risk after the outcome has occurred, the logistic regression in our study also represents a Cox proportional hazard regression. The odds ratios form weighted pooled logistic regression were the hazard ratios. Explanatory variables included baseline data and initial tuberculosis, and response variables included ART. Huber-White estimated the standard error of the variables. 

We first studied the association between baseline data, ART and death. The same processes were repeated among patients with initial CD4 cell counts of 350 cells/µL or less. A marginal structural mode then demonstrated the associations between ART, death and tuberculosis by inverse probability weights. The above analysis was completed by Stata 12.0 (StataCorp, College Station, TX, USA).

## 3. Results

By February 2014, 6897 People Living with HIV (PLHIV) had been enrolled and followed up for a maximum of 178 months (median, 15 months; IQR, 6–30 months) after enrollment. Of these, 3098 (44.92%) received ART for up to 178 months (median, 20 months; IQR, 8–33 months) after enrollment. The median number of contacts with a physician or nurse was 26 (IQR, 19–36) among patients who received ART and 11 (IQR, 5–17) among patients without ART. A total of 3,815 patients (55.31%) had initial CD4 cell counts less than 350 cells/µL, and 2674 of these (70.09%) initiated ART during the study period (Table 1). Among patients with initial CD4 cell counts less than 350 cells/µL, those who were older, female, native, baseline tuberculosis positive, baseline AIDS stage and had a lower initial CD4 cell count were more likely to receive ART. Those who were not married or had a history of drug use were more likely to not receive ART (*p* < 0.001).

Among all patients who received ART, there were 98 deaths (3.16%), 59 new tuberculosis diagnoses (1.90%), and 6097 person-years observed after commencement of ART. Among those not who did not receive ART, there were 410 deaths (10.79%), 201 new tuberculosis diagnoses (5.29%), and 5634 person-years observed. Therefore, the crude incidence rate ratios associated with ART were 0.22 (95% CI, 0.18–0.28) for death and 0.27 (95% CI, 0.20–0.37) for tuberculosis. Among a subset of patients with initial CD4 cell counts less than 350 cells/µL who received ART, 79 deaths (2.95%) and 30 new tuberculosis diagnoses (1.12%) were observed in 5360 person-years after initiating treatment; among the subset of patients with initial CD4 cell counts less than 350 cells/µL who did not receive ART, 151 deaths (13.23%) and 119 tuberculosis diagnoses (10.43%) were observed in 1426 person-years. Therefore, the crude incidence rate ratios associated with ART among patients in this subset were 0.14 (95% CI, 0.11–0.18) for death and 0.07 (95% CI, 0.05–0.10) for tuberculosis.

**Table 1:** Baseline Characteristics of HIV/AIDS Patients in Shenzhen, China from December 2003 to February 2014.

Characteristic	Patients with Baseline CD4 Cell Count Less Than 350 cells/μL				
	All Patients (N = 6897)	All Patients (N = 3815)	No ART (N = 1141)	ART (N = 2674)	*P*
Median Age	32 (IQR, 27–39)	33 (28–40)	32 (26–39)	33 (28–41)	<0.001
Gender					<0.001
Male	5768 (83.63%)	3105 (81.39%)	984 (86.24%)	2121 (79.32%)	
Female	1129 (16.37%)	710 (18.61%)	157 (13.76%)	553 (20.68%)	
Native Resident					<0.001
Yes	4841 (72.19%)	2826 (74.08%)	739 (64.77%)	2087 (78.05%)	
No	2056 (27.81%)	989 (25.92%)	402 (35.23%)	587 (21.95%)	
Marital Status					<0.001
Unmarried	3707 (53.75%)	1863 (48.83%)	649 (56.88%)	1214 (45.40%)	
Married	2158 (31.29%)	1370 (35.91%)	312 (27.34%)	1058 (39.57%)	
Divorced	963 (13.96%)	564 (14.78%)	173 (15.16%)	391 (14.62%)	
Unknown	69 (1%)	18 (0.48%)	7 (0.62%)	11 (0.41%)	
Infection Route					<0.001
Heterosexual	3686 (53.44%)	2166 (56.79%)	614 (53.81%)	1552 (58.04%)	
Homosexual	2194 (31.81%)	1186 (31.09%)	337 (29.54%)	849 (31.75%)	
Injection Drug Use	775 (11.24%)	334 (8.75%)	165 (14.46%)	169 (6.32%)	
Others	222 (3.22%)	121 (3.17%)	22 (1.93%)	99 (3.70%)	
Unknown	20 (0.29%)	8 (0.20%)	2 (0.18%)	6 (0.22%)	
Baseline AIDS Stage					<0.001
Yes	2803 (40.64%)	2409 (63.15%)	409 (35.85%)	2000 (74.79%)	
No	4094 (59.36%)	1406 (36.85%)	732 (64.15%)	674 (25.21%)	
Baseline Tuberculosis					<0.001
Positive	310 (4.49%)	210 (5.50%)	30 (2.63%)	180 (6.73%)	
Negative	6587 (95.51%)	3605 (94.50%)	1111 (97.37%)	2494 (93.27%)	
Baseline CD4 Cell Count, cells/µL					<0.001
0–99	996 (14.44%)	996 (26.11%)	224 (19.63%)	772 (28.87%)	
100–199	894 (12.96%)	894 (23.43%)	116 (10.17%)	778 (29.09%)	
200–349	1925 (27.91%)	1925 (50.46%)	801 (70.11%)	1124 (42.07%)	
≥350	2441 (35.39%)		-	-	
Unknown	641 (9.30%)		-	-	

Abbreviation: ART, antiretroviral therapy.

**Figure 1:**
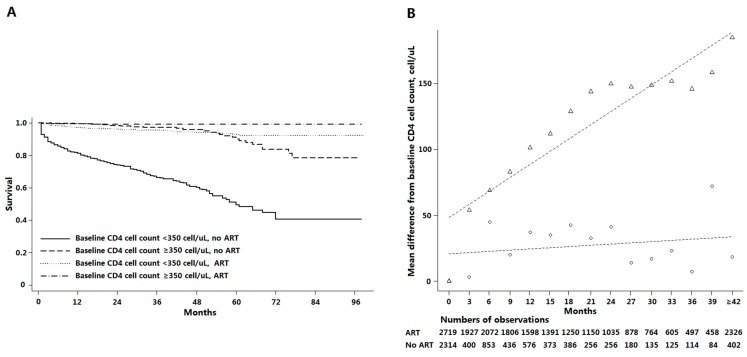
Effectiveness of antiretroviral therapy on survival and changes in CD4 cell counts. (**A**) Kaplan-Meier survival estimates by baseline CD4 cell count and by treatment status. For patients receiving antiretroviral therapy (ART), time is months since commencement of therapy. For patients without ART, time is months since enrollment in this study. (**B**) Changes in CD4 cell counts over time as percentages of baseline CD4 cell counts. For patients receiving ART (triangle), time is months since commencement of therapy. For patients without ART (circles), time is months since enrollment in this study. The dashed lines are a fitted regression line for these patients.

After adjusting for other covariates, patients were more likely to receive ART if they were older, female, had a lower initial CD4 cell count, had tuberculosis or were AIDS stage at baseline (Table 2). The dramatic effect of ART on survival among patients with initial CD4 cell counts less than 350 cells/µL is shown in Figure 1A. The hazard of death was higher if patients had lower baseline or latest CD4 cell counts, received ART, or had tuberculosis at baseline (Table 2). In the marginal structural models that included all patients, the hazard ratios for death among those receiving ART were 0.18 those of patients not receiving ART (Table 3).

**Table 2:** Factors Associated With Starting ART or Death.

	ART		Death	
	Hazard Ratio(95% *CI*)	*P*	Hazard Ratio(95% *CI*)	*P*
Age Group, year				
16–25	1.00	-	1.00	-
26–35	1.60 (1.26–2.02)	<0.001	1.13 (0.59–2.18)	0.699
36–45	2.05 (1.56–2.68)	<0.001	1.41 (0.70–2.85)	0.338
>45	2.61 (1.81–3.77)	<0.001	1.87 (0.83–4.19)	0.129
Sex (Female *vs.*Male)	2.35 (1.77–3.12)	<0.001	0.97 (0.52–1.81)	0.916
Baseline CD4 Cell Count, cells/µL				
0–99	1.00	-	1.00	-
100–199	0.35 (0.22–0.58)	<0.001	0.21 (0.14–0.31)	<0.001
200–349	0.19 (0.12–0.31)	<0.001	0.13 (0.09–0.19)	<0.001
≥350	0.02 (0.01–0.03)	<0.001	0.10 (0.07–0.14)	<0.001
Latest CD4 Cell Count, cells/µL				
0–99	-	-	1.00	
100–199	-	-	0.13 (0.07–0.24)	<0.001
200–349	-	-	0.05 (0.02–0.10)	<0.001
≥350	-	-	0.03 (0.02–0.08)	<0.001
Baseline AIDS Stage (Yes *vs.*No)	16.40 (12.31–21.86)	<0.001	1.79 (0.81–3.94)	0.148
Baseline Tuberculosis (Yes *vs.*No)	1.81 (1.06–3.07)	0.029	1.63 (1.16–2.08)	0.004
ART (Yes *vs*.No)	-	-	0.21 (0.12–0.35)	<0.001

Abbreviation: ART, antiretroviral therapy; Pooled unweighted logistic regression models also adjusted for location and months of follow-up.

**Table 3:** Estimated Effects of ART from Unweighted and Weighted Cox Models.

Variable	Outcome	Unweighted Models, No Covariates	Unweighted Models, Baseline and Time-Varying Covariates	Weighted Model Baseline Covariates ^a^	Patients (N)
Patients with any baseline CD4 cell count					
ART	Death	0.25 (0.19–0.38)	0.21 (0.14–0.31)	0.18 (0.11–0.27)	6897
ART	Tuberculosis	0.34 (0.26–0.43)	0.29 (0.21–0.38)	0.27 (0.19–0.37)	6897
Per additional month of ART	CD4 cell count, cells/µL	5.67 (5.19–6.01)	5.83 (5.29–6.41)	6.52 (6.08–7.12)	2719 ^b^
Patients with baseline CD4 cell count less than 350 cells/µL					
ART	Death	0.21 (0.15–0.29)	0.15 (0.12–0.19)	0.10 (0.06–0.13)	3815
ART	Tuberculosis	0.15 (0.11–0.20)	0.09 (0.06–0.11)	0.06 (0.03–0.08)	3815
Per additional month of ART	CD4 cell count, cells/µL	10.21 (9.32–11.01)	4.52 (4.19–4.92)	5.23 (4.91–5.59)	1432 **^b^**

Abbreviation: ART, antiretroviral therapy; ^**a**^: Weighted model with baseline covariates estimates parameters of marginal structural model; ^**b**^: Patients with at least 2 CD4 cell count measurements and receiving ART.

Crude trends in CD4 cell count responses are compared in Figure 1B for patients who did or did not receive ART. Among the CD4 cell count change models, linear time models showed that CD4 cell counts increased steadily during ART. But owing to CD4 cell counts recovering to relatively high level, there was a plateau between 24 to 36 months. And considering fewer patients in follow-up, the increasing level of CD4 cell counts fluctuated after 36 months. Marginal structural models that included all patients demonstrated that for each extra month of treatment since initiating ART, CD4 cell counts increased by 6.52 cells/µL (Table 3). 

Secondary analyses demonstrated similar effect estimates. As expected, estimates of the effect of ART from weighted models were stronger than those from unweighted models (Table 3). In the subgroup of patients with an initial CD4 cell count less than 350 cells/µL, ART had a stronger effect on mortality (HR = 0.10, 95% CI = 0.06–0.13) and the presence of tuberculosis (HR = 0.06, 95% CI = 0.03–0.08), and a weaker effect on CD4 cell count increase per month (5.23 cells/µL, 95% CI = 4.91–5.59). 

## 4. Discussion and Conclusions

The study enrolled 6897 adult patients with HIV infection to estimates the effects of ART on mortality, newly-diagnosed tuberculosis, and CD4 cell counts in Shenzhen in a ten-year observational cohort study. Because most HIV-infected patients starting ART will not have been treated previously, it is required that estimates of its effectiveness should be compared with no treatment, especially in view of possible adverse effects. The outcomes of HIV-infected patients in China have substantially improved since 2003 through the widespread use of potent antiretroviral therapy [6]. The extensive benefits of ART [14,15] provide a framework for the debate about the relative cost-effectiveness of treatment compared with prevention in developing areas, such as China and sub-Saharan Africa.

Our findings show a strong relation between ART and HIV-related mortality, in accordance with the results of previous studies in China and other countries [10,12,16,17]. Thus, the mortality of HIV-infection patients may be decreased effectively by increasing coverage and commencing ART. Although the State Council formed an AIDS Working Committee, and the Four Frees and One Care policy was issued ten years ago, China’s treatment coverage of 63.4% across 2513 treatment sites indicates there is a long way to go to achieve the goal of complete coverage of people who meet treatment criteria [18]. Policies such as the NFATP were initially piloted and scaled up in populations of former plasma donors, and previous studies in China focused on the effect of ART in former plasma donors [9]. However, with lower treatment coverage and higher mortality, patients infected through sexual transmission or drug use need more screening and treatment [5,19]. Actually, the proportion of homosexual patients in our study may not reflect the actual proportion of diagnosed cases, because many MSM patients may conceal their information of sexual orientation in China.

The other risk factor strongly related to death was delayed treatment initiation. Many studies have shown increased mortality in patients whose CD4 cell counts are low at baseline [20,21] and late diagnosis leads to delayed treatment initiation [22]. Despite efforts to screen high-risk populations in China for HIV, individuals with infection are still diagnosed late in their disease course. Therefore, it is necessary to consider the urgent expansion of the WHO-recommended provider-initiated testing and counseling strategy to high-risk cohorts or locations with a known higher prevalence of HIV. In China, high-risk populations in which provider-initiated testing and counseling could be implemented include not only injection drug users, female sex workers, and men who have sex with men, but also individuals attending clinics for tuberculosis, sexually transmitted disease, and antenatal care, and those in counties with an HIV prevalence above 1%.

Our ten years of follow-up among HIV-infected patients selected as being most ready to receive ART can be seemly extended to larger-scale provisions or to longer-term treatment. A strength of this study was our use of marginal structural models to estimate ART effectiveness by adjustment for time-varying CD4 cell counts in order to avoid selection bias. Since time-dependent confounders affected by treatment were not accurately taken into account, previous analyses of observational studies may have underestimated the effect of ART [5,9]. In this study, as patients with lower counts were more likely to be treated, CD4 count was a time-dependent confounder for the effect of ART. CD4 count is also affected by ART, and was thus intermediate on the causal pathway from treatment to death. In this situation, standard approaches such as Cox regression will yield biased estimates of the effect of treatment [23,24]. A comparison of weighted and unweighted models (Table 3) suggests that unweighted models underestimated the effects of ART on death, tuberculosis and CD4 cell count.

Treatment efficacy also may have been underestimated by assuming that patients continued to receive ART once started. Since antiretroviral therapy is free in China, patients will not remain untreated for economic reasons, so few patients interrupted ART. By the end of follow-up, 2.48% of patients had received ART for less than three months. Noncompliance would not have biased the estimated effect on outcomes of tuberculosis and CD4 cell count because mortality was tracked even if patients defaulted, whereas other analyses were censored at the last measurement or visit. This was also another strength of our study.

We also may have underestimated the incidence of tuberculosis, especially among patients not receiving ART who were seen less often. Therefore, the effect of ART on tuberculosis was probably underestimated. But tuberculosis at baseline was associated with receiving ART and increasing the risk of death. The reason may be that patients with tuberculosis at baseline may be at a critical stage of HIV/AIDS and more likely to receive ART.

The lack of data on viral loads and WHO stage for untreated patients was a limitation. Due to constraints of costs and technology, a standard practice of monitoring plasma viral load that is often performed in developed countries was not recommended in low-income countries. Viral load testing could be increasingly important in guiding clinical decisions aside from selecting treatment [25]. In addition, little data was received on patients enrolled during the first two years, because the recording of CD4 cell counts was scaled up after about 2005 in China [13]. However, the proportion of patients enrolled before 2005 was very small. In addition, in accordance with Chinese policy, ART was given to all HIV-positive patients who met the national treatment guidelines of a CD4 cell count less than 200 cells/µL or WHO stage 3 or 4 disease before 2008. But after 2008, this policy was changed and patients with a CD4 cell count less than 350 cells/µL should receive ART. In our study, Patients were collected from December 2003 to February 2014. So, only 70% of those qualifying for ART initiated treatment.

In conclusion, although China’s national HIV program has made much progress during the past ten years, most notably in the successful diagnosis, treatment and resultant decreased mortality, there is an urgent need for large-scale HIV screening of high-risk populations, and increasing coverage and timely commencement of ART.
